# Job Mobility and Wealth Inequality

**DOI:** 10.1007/s10614-020-10064-8

**Published:** 2020-10-30

**Authors:** J. M. Applegate, Marco A. Janssen

**Affiliations:** 1 Complex Systems Research Group, Arizona State University, 1031 South Palm Walk, Tempe, AZ 85281 USA; 2 School of Sustainability, School of Complex Adaptive Systems, Arizona State University, 800 Cady Mall, Tempe, AZ 85281 USA

**Keywords:** Emergence, Job mobility, Inequality, Wages, Household debt, Firm productivity, C61, D63, G51, J62

## Abstract

The extent to which employees change jobs, known as the job mobility rate, has been steadily declining in the US for decades. This decline is understood to have a negative impact on both productivity and wages, and econometric studies fail to support any single cause brought forward. This decline coincides with decreases in household savings, increases in household debt and wage stagnation. We propose that the decline could be the consequence of a complex interaction between mobility, savings, wages and debt, such that if changing jobs incurs costs which are paid out of savings, or incurs debt in the absence of sufficient savings, a negative feedback loop is generated. People are further restricted in making moves by their debt obligations and inability to save, which in turn depresses wages further. To explore this hypothesis, we developed a stylized model in which agents chose their employment situation based on their opportunities and preferences for work and where there are costs to changing jobs and the possibility of borrowing to meet those costs. We indeed found evidence of a negative feedback loop involving changes, wages, savings and debt, as well as evidence that this dynamic results in a level of wealth inequality on the same scale as we see today in the US.

## Introduction

The US job mobility rate, describing the extent to which employees move between employers, is at an all time low after declining for decades, and this decline has important consequences. Job changing is understood to improve productivity by matching workers to more suitable employment and by promoting innovation through the inter-firm exchange of experience (Eriksson and Lindgren 2008; Helsley and Strange 1990; Breschi and Lissoni 2009). Changing jobs is also understood to increase wages by providing workers with opportunities to negotiate higher salaries (Gottschalk 2001).

Why has job changing become less frequent over the past several decades? Suggested causes include a need to retain employer-provided health insurance, an aging population, the rise of dual-career households, declining entrepreneurship, a decline in middle-skill jobs, burdensome occupational licensing requirements or skill supply and demand mismatches. Yet econometric studies do not provide strong support for any of these explanations (Hyatt 2015; Molloy et al. 2017).

Another possible explanation is that changing jobs incurs costs on the part of the employee, such as gaps in income, training expenses or relocation costs, and these costs are funded by the employee by spending savings or borrowing through loans [also suggested by (Bhaskar et al. 2002)].^1^ A broad exploration of the employee cost burden in the reallocation process is missing from the literature, perhaps in part because of the difficulty in quantifying such costs.

A further consideration is that this decline could be the result of a complex interaction of several factors. The decrease in job mobility is contemporaneous with decreases in household savings (Guidolin and La Jeunesse 2007), increases in household debt (Getter 1996), and a stagnation of wages (Donovan and Bradley 2018).^2^ Could a decrease in savings and an increased debt burden be impacting the ability of workers to take advantage of wage and productivity improving job opportunities, thus further impeding their ability to accrue savings?

Some evidence suggests this could be the case. Owing more on a mortgage than the market value of the house has an impact on job mobility, to the extent that people take lesser jobs in order to avoid the costs of moving (Brown and Matsa 2016)^3^. The decline in US savings rates strongly correlates with increased credit availability (Carroll et al. 2019), suggesting that households are substituting debt for savings. Barba and Pivetti claim evidence of the substitution of loans for actual wages (2008), further supported by findings of sharp increases in the use of consumer credit applied to necessitous spending, where households borrow to make regular purchases, which in turn may lead to liquidity traps that make future saving difficult (Pollin 1988; Sullivan et al. 2001; Weller 2007; Eggertsson and Krugman 2012).^4^ Could mobility, wages and debt interact to generate a negative feedback loop, which differentially applied across a population, be one of the mechanisms driving wealth inequality?

Informed by the evidence presented above, we propose that if pursuing improving work opportunities requires some amount of financial capital, then individuals without savings either miss out on wage increasing opportunities or resort to borrowing, which impedes their future ability to save, and that this dynamic may be a driver of wealth inequality.^5^^,^^6^

Thus we wish to explore a complex interaction between savings, lending and wages to explain job mobility and its consequences for wealth. Kirman (2011) defines economic complexity as agent interactions generating phenomena at the macroeconomic level that do not coincide with observations at the microeconomic, so in that spirit we have developed a stylized multi-agent model, the Emergent Firms (EF) model, to explore the emergent effects of individual work choices in the context of job change costs, savings and lending.

We indeed find that if pursuing a job opportunity incurs costs, then having financial capital matters, and without it, and especially in the presence of debt, agents are limited in their ability to fully participate in the stylized economy. The strength of the relationships found in the model may generate testable hypotheses (Griffin 2006) as well as justify efforts to seek techniques and datasets to demonstrate these complex feedback effects more explicitly.

## The Emergent Firms Model

The EF model is based on Rob Axtell’s *Endogenous Dynamics of Multi-Agent Firms Model*, where agents chose their employment situation based on their opportunities and preferences for work (1999; 2015; 2018; 2019). The intent of the Axtell model is to describe the overall distribution of firm sizes as the emergent property of numerous individual choices about where to work. Therefore, the Axtell model is also a job mobility model, and as such provides a uniquely suitable starting point for an exploration of the effects of costs, savings and credit on mobility dynamics.^7^

The EF model is driven by an agent’s choice to work with other agents in order to advantage itself of the benefits of returns-to-scale and coordination. Numerous agents explore options for changing firms, becoming self employed as a *singleton* firm, or remaining in their current position. Agents chose their best option based on maximizing a Cobb-Douglas utility function with their individual preference set for income and leisure,

1 $$\begin{aligned} U = \left( \frac{O}{n}\right) ^\theta (\omega - e)^{1 - \theta }, \end{aligned}$$

where *O* is total firm output, *n* the number of agents in the firm, such that $\frac{O}{n}$ is the agent’s income in the current firm configuration. The agent’s preference for income is given by $\theta$, therefore preference for leisure is $1 - \theta$. The agent’s total time endowment is $\omega$ and *e* is the agent’s work effort, thus the agent’s leisure is $\omega - e$.

Each firm adopts its founder’s values for *a*, *b* and $\beta$ which characterize the returns to scale in the firm’s production function, thus each firm will have differing production capabilities which could represent differences in production technology or in the managerial ability to appropriately utilize employees’ skills. The firm’s output is divided evenly between all employee agents, and each agent’s portion of the output is its wage. Therefore an agent’s wage is not only a function of its own effort, but of the positive returns to scale obtained by combining its efforts with other agents, and an agent could obtain very different wages for the same effort depending on the configuration of its employing firm.

2 $$\begin{aligned} O = aE + bE^\beta , \end{aligned}$$

where *E* is the sum of all the firm agents’ efforts.

Agents are characterized by both preferences for income and savings rates, as well as by the production function parameters that determine firm output levels. Agents are also connected via an underlying social network, modeled as an Erdös-Renyi network, and can choose to join a firm that employs a neighbor in this social network (Montgomery 1991).^8^ The founder of a firm determines the production function parameter values for that firm.

These basic microeconomic principles embodied by a group of utility seeking agents create macroeconomic conditions of “fluctuating effort and sustainable cooperation” (Huberman and Glance 1998), and provide the engine of free movement job mobility. Agents choose an optimal individual effort to maximize their utility, given the output of a combined effort (*E*) of all agents in the firm. Therefore, the optimal effort for an agent will decrease as other agent efforts in the same firm increase, thus creating a free-rider problem. As free-riding increases, the utility for other firm members decreases, such that an optimal firm size that maximizes wages is not the same as one that maximizes utility. Thus agents will leave firms with high wages if they find higher utility elsewhere, even if their resulting wages are lower.^9^

The Axtell model approach differs from other agent-based models exploring wage dynamics such as those of Dawid and Genkow (2014) or Dosi (2018) in that Axtell does not model a closed-system production-based cycle, where firm output is determined by consumer and producer demand, which in turn determines wages. Agents, in this and the Axtell model, spend the portion of their wage not saved, but the source of the consumption goods agents spend on and the destination of goods produced by firms is not considered as relevant in the context of the public goods game. Wages are assumed to rise with increased production, the shared output from the firm is considered a wage rather than a divided, and wages are not determined through a labor market.

The model dynamics also make no recourse to innovation or investment, rather firms are founded when an agent’s optimal choice is to become self employed. Self employment can lead to a multi-employee firm if the founding agent attracts others to work with it. Although a large portion of self employed persons are not entrepreneurs and don’t intend to grow their firms, we continue with this assumption that individuals who choose self employment have the potential to become firm founders with employees (Hurst and Pugsley 2011). Futhermore, we assume no technological limitations on economies of scale.

As explained in Sect. 1, job changes may incur costs and we are seeking to explore what effects the presence of costs has on job mobility. We further assume costs exceed those that could be regarded as general household expenditures, that these costs are not smoothed over a period of time, and are funded via savings or borrowing (Sullivan 2008; Clark and Davies Withers 1999). The ability to make a change will therefore be dependent on an agent’s savings and access to credit. Therefore we add two components in a gradual manner to create two new scenarios.

The first addition applies costs for employment changes, with a cash-in-advance constraint which means an agent must have available funds to make a change (Lucas and Stokey 1985). Agents save a portion of their wage each time step, the quantity dependent on their individual saving rate, and savings accrue until agents spend all or a portion on making an employment change. We assume living costs are covered by wage and any residual goes into savings, so the varied savings rates are a proxy for varied levels of consumption. There are two aspects of mobility costs that need to be considered from the modeling perspective; the heterogeneity of costs and the level of costs. We model the costs of changing jobs as specific to each agent to provide heterogeneity, and for simplicity we consider the level of costs as a linear function of an agent’s current employment, which explicitly accounts for effort preferences and implicitly for a skill set or network connections. This is the cost scenario.

The second addition adds to the costs scenario a universal credit-creating lender who makes funds available to agents with insufficient savings to make a move. The financial economy and the real economy are treated as decoupled systems such that loanable funds are not sourced from the savings of other agents (Minsky 1992; Mehrling 2010; Werner 2014; Jakab and Kumhof 2015). Loans incur interest compounded each time step at a constant rate, and are paid with a borrowing agent’s full savings each step until repaid. It is not our intent to model loan dynamics intensively, rather to explore the effects of debt on job mobility. Loans are made based on current income to those agents without existing loans, which mimics lender selectivity, and there is no bankruptcy option for indebted agents. This is the costs with credit scenario.

**Fig. 1:**
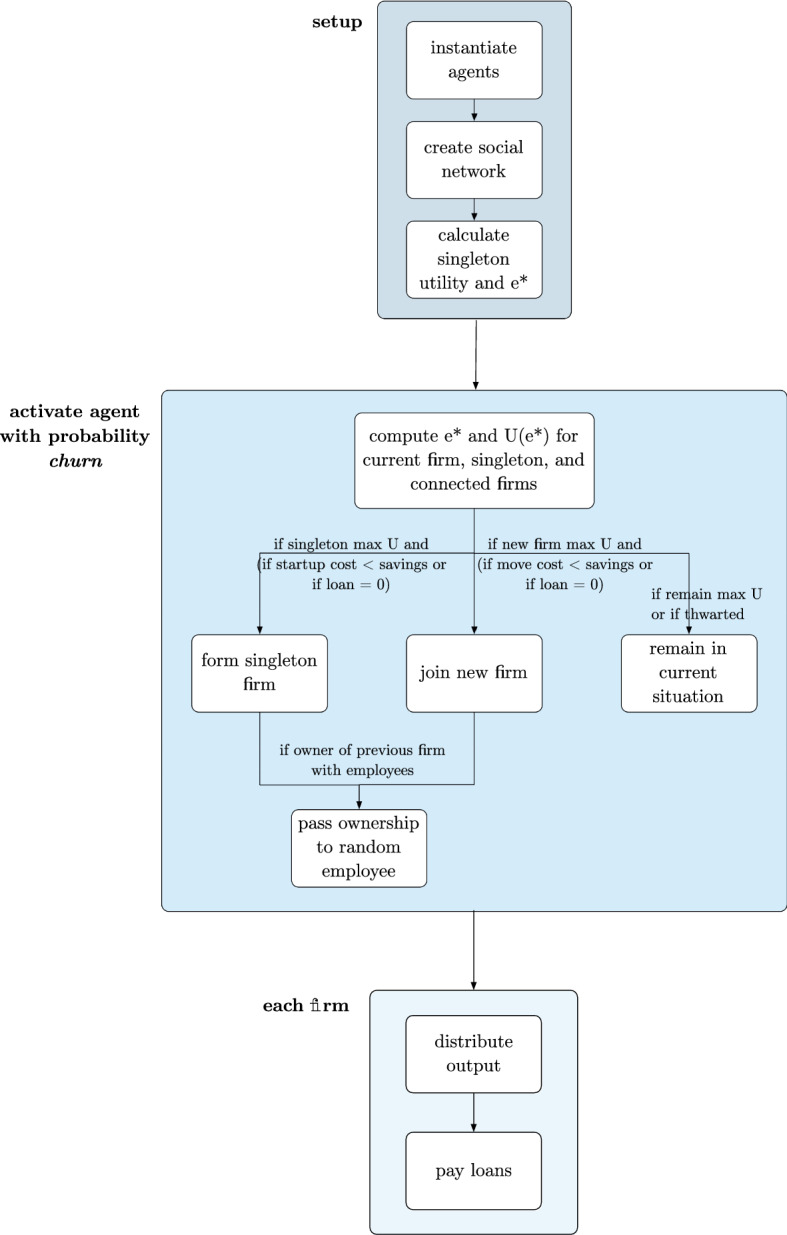
Algorithmic flow for the emergent firm model

The full EF model functionality is illustrated as a flowchart in Fig. 1. Cost and lending functionality can be toggled independently so we can explore three distinct scenarios: free movement, costs, and costs with credit.^10^

Experiments with the EF model were made over 30 runs for 600 agents over 500 steps with an activation rate, or *churn*, of 10%. Therefore an average of 60 agents explore alternative employment options each step, for a total of 30,000 explorations for each of the 30 simulation runs. Sensitivity analyses demonstrate a stability of results for the base model at these values. Table 1 describes the parameters in the EF model and their values.

**Table 1:** Model parameters

Attribute	Description	Value
a	Effort multiplier in output formula	$\mathcal {U}(0, .5)$
b	Exponential effort multiplier	$\mathcal {U}(.75, 1.25)$
$\beta$	Returns to scale exponent	$\mathcal {U}(1.5, 2)$
$\theta$	Preference for income	$\mathcal {U}(0, 1)$
$\omega$	Time endowment	1
$\nu$	Number of social network links	$\mathcal {U}(2, 6)$
	Compensation rule	equal shares
	Initial condition	all singleton firms
N	Number of agents	600
churn	Agent activation rate	.1
tmax	Number of steps	500
move	Job change cost, multiplies last wage	1
self employment	Self employment cost, multiplies last wage	2
rate	Multiplies wage each time step	$\mathcal {N}(.03, .01),$ truncated at 0
lendingrate	Cost of loan each time step	.03

$\mathcal {U}$ and $\mathcal {N}$ indicate random draws form the uniform distribution with (min, max) or the normal distribution with (mean, standard deviation) The base model results in this paper were run with a random seed

## Results

For the three scenarios described in Sect. 2 we explored the number of employment changes and missed opportunities for change (described as *thwarts*), wages, firm productivity, loans and debts, as well as savings and total wealth. The institutional conditions represented by the costs and the costs with credit scenarios implemented at the microeconomic level have statistically significant effects on these macroeconomic measures in their respective emergent economies. Unless otherwise indicated, all simulations were run 30 times with the parameters and settings described in Table 1.

**Fig. 2:**
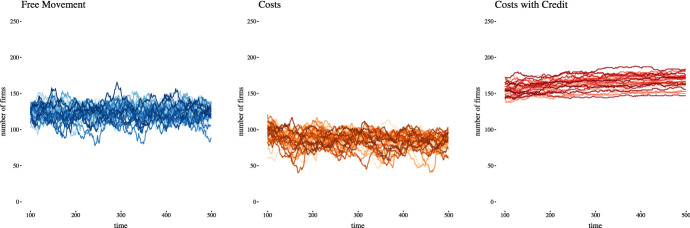
Macroeconomic convergence of total number of firms. Spaghetti plot of number of firms over time showing firm population results for 30 distinct runs for all three scenarios, starting at $t = 100$ and demonstrating the macroeconomic convergence into a steady state equilibrium band. Notice the downward and upward trends in the costs and costs with credit scenarios, and the relative volatilities within the bands

The emergence of regions of steady state firm population stability, even though the composition and size of any given firm is in flux, is common across the scenarios. This equilibrium region emerges after roughly 100 time steps and the number of firms oscillates within a band, as demonstrated in the 30 run spaghetti time series plots for each scenario in Fig. 2.^11^ By 500 steps the band for the free movement scenario is level, while the costs scenario demonstrates a slight downward trend ($-\,3\%$) and the costs with credit scenario a slight upward trend in this equilibrium band ($+\,3\%$).

The costs with credit scenario produces the most firms, therefore the smallest firms. The costs scenario produces the fewest, conversely the largest, firms and the number of firms in the free movement scenario falls between the two. Underlying these firm populations dynamics are employment dynamics, and the following sections explore aspects of job mobility in the contexts of free movement, costs, and costs with credit.

### Mobility: Changes and Thwarts

**Fig. 3:**
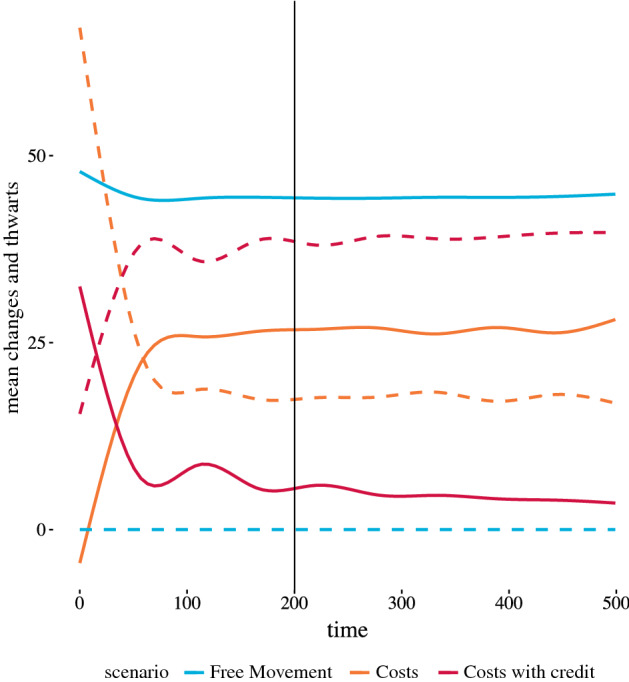
Mobility measures over time for three scenarios. Generalized additive model fits for the two mobility descriptors: changes and thwarts, with total changes (solid lines) and thwarts (dashed lines) over time for 30 runs and the three scenarios, indicated by color

An agent’s mobility describes its ability to make a desired change, and agents with lower mobility miss out on opportunities more often than agents with higher mobility. Figure 3 shows a generalized additive model fit of the total number of employment changes and thwarts across 30 runs for each scenario. Mean numbers of changes and thwarts for the three scenarios at ${\texttt {t}} = 500$ are 45 changes and 0 thwarts for the free movement scenario, 28 changes and 16 thwarts for the costs scenario, and 4 changes and 39 thwarts for the costs with credit scenario, though we notice for this scenario the changes are continuing downward and thwarts upward. There are no restrictions on making changes in the free movement scenario, so any utility improving opportunity can be acted upon, thus the number of thwarts is 0 and this scenario consequently produces the greatest number of changes.

The number of changes decreases across the scenarios and conversely the number of thwarts increases. Since the costs scenario produces more changes than the costs with credit scenario, we find the counterintuitive result that being able to borrow to make a move in aggregate results in fewer moves than overcoming the cost constraint via savings alone. As the level of costs increase, thwarts increase as well. We ran sensitivity analysis with various values for the wage multiplier, as well as modeling costs as a function of expected future wages rather than current wages. (Results for the change in cost scheme is found in “Appendix A.2.”) Basing costs on expected future wages produced results similar to increasing the cost multiplier, and the observed patterns across the three scenarios is the same: fewer changes for the costs scenario compared to the free movement scenario, and even fewer changes for the costs with lending scenario. As the cost level rises, differences in the changes across scenarios become more pronounced.

Statistical summaries of select macroeconomic variables including changes and thwarts across the scenarios are given in Table 2.

**Table 2:** Means and standard deviations for select macroeconomic measures

Measure	Free movement	Costs	Costs with credit
Number of firms	124.87 (10.38)	84.67 (11.04)	166.80 (9.32)
Changes	45.13 (5.89)	28.03 (6.20)	3.67 (1.85)
Thwarts	0 (0)	16.10 (4.80)	38.93 (6.27)
Wage	.56 (.11)	.59 (.08)	.53 (.03)
Firm productivity	2.77 (.90)	4.33 (1.15)	1.93 (.19)
Savings	8.56 (.81)	1.11 (.23)	1.29 (.17)

Means and standard deviations for scenario measures for all agents and all runs at the final time step (t = 500). Equality of means tests for each combination of scenarios for each measure were conducted via the Welch Two Sample t-test, with all differences found to be highly significant (*p*-values $\simeq 0$)

### Wages and Firm Productivity

Wages are an employee’s share of firm output, as defined in Eq. 2, distributed each time step, and a firm’s productivity is synonymous with the firm’s output.^12^ Mean wages for the three scenarios are on average .56, .59 and .53, respectively, all significantly different across scenarios according the Welch t-tests. Summary statistics are given in Table 2 and note that the costs with credit scenario produces not only the lowest wages but also the least variation in wages. Over time, wage values in the free movement and the costs scenarios oscillate, while wages in the cost with credit scenario continuosly decline.

The mean firm productivity values for the three scenarios are 2.77, 4.33 and 1.93, with the costs scenario producing the most productive firms and the costs with credit scenario the least productive firms. This is a consequence of the cost scenario firms being larger, so fewer of them, and wages highest, while the lowest wages and the most firms are produced by the costs with credit scenario.

One of the effects of agents setting effort according the utility function described in Eq. 1 is that as wages decrease, so does effort. This decrease is wage due to decreased effort further decreases an agent’s effort until they find a better situation, and results in a portion of agents with wage values near zero.

### Agent Parameter Correlations with Firm Size and Wage

Firm sizes and wages emerge out of the interactions of utility maximizing agents who have differing preferences for income over leisure, given by $\theta$, and who form firms with production characteristics, *a*, *b* and $\beta$, determined by the founding agent. For firm sizes greater than 1, meaning all firms that are engaged in co-production, Table 3 shows correlations for relationships between founding agent parameters and wage or firm size where at least one scenario correlation value is greater the .25, for each of the scenarios. We have also broken down the relationships for the costs with credit scenario, dividing the population into three categories; those with increasing debt, those with decreasing debt, and those with no debt at t = 500. These costs with credit subpopulation results are shown in Table 4.

**Table 3:** Wage and size correlations

Scenario	Wage, size	Wage, $\theta$	Size, $\beta$
Free movement	.25	.23	.36
Costs	.18	.18	.43
Costs with credit	.03	.19	.28

Correlations between wage, size, income preference ($\theta$) and production returns to scale ($\beta$) for the three model scenarios

**Table 4:** Costs with credit scenario subpopulation values and correlations

Subpopulation	Count	Mean $\theta$	Mean $\beta$	Wage, size	Wage, $\theta$	Size, $\beta$
No debt	4622	.35	1.78	− .08	.37	.28
Decreasing debt	12076	.56	1.74	.09	.29	.26
Increasing debt	1302	.43	1.73	− .05	.31	.37

Statistics and correlations between wage, size, income preference ($\theta$) and production returns to scale ($\beta$) for three subpopulations in the costs with credit scenario. Values for mean $\theta$ and $\beta$ are statistically different according to t-tests. The counts sum to 18000, which are 600 agents over 30 runs

Wage and size are slightly correlated in the free movement scenario, less correlated in the costs scenario, and uncorrelated in the costs with credit scenario (Table 3). Wage and $\theta$ are slightly correlated in the free movement scenario, and less correlated in the costs and the costs with credit scenarios. Size and firm $\beta$ are lightly correlated in each of the scenarios, with the strongest correlation in the costs scenario, and the weakest correlation in the costs with credit scenario. When the costs with credit scenario is divided into subpopulations, the correlations between wage and $\theta$ increase, mostly for the population with no debt, and the wage size correlations disappear (Table 4). The correlation between firm size and firm $\beta$ strengthens for the subpopulation of agents with increasing debt. Additionally, the decreasing debt subpopulation has the highest mean $\theta$, the debt-free population has the lowest mean $\theta$ and the highest mean firm $\beta$, and the increasing debt population has the lowest mean firm $\beta$.

### Savings and Debt

**Fig. 4:**
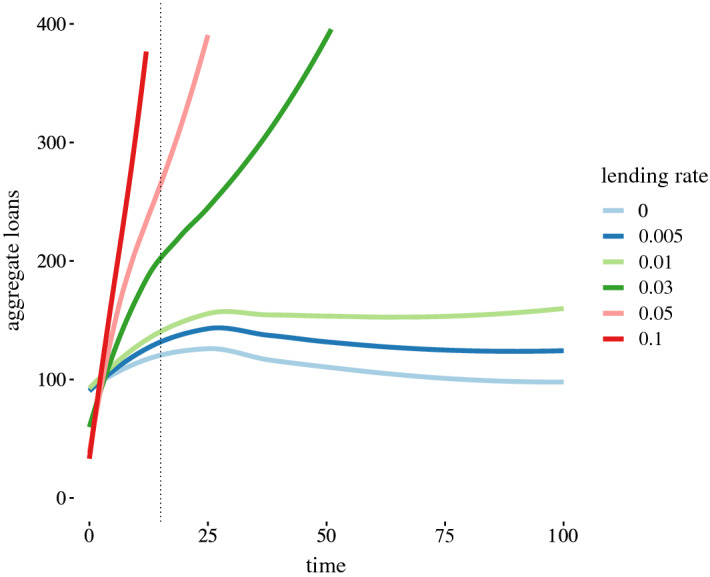
Total loan amounts by lending rate for 30 runs. Note that 1% and .5% have just begun their upward curve, which increase in slope after ${\texttt {t}} = 100$. The larger the interest rate the faster the superlinear behavior manifests

All agents have a non-zero savings rate, so will save a percentage of their wage. The costs with credit scenario allows agents with insufficient savings to pursue utility improving opportunities by taking out a loan. Debt quickly becomes pronounced, increasing superlinearly for lending rates above 0, as demonstrated in Fig. 4. The superlinear loan behaviour for model simulations (an interest rate of 3%) starts around ${\texttt {t}} = 15$. In this scenario, all indebted agents’ savings goes to servicing debt, which results in those agents no longer accruing savings until the debt is paid. The superlinear growth in loans manifests as agents are unable to repay loans.

**Fig. 5:**
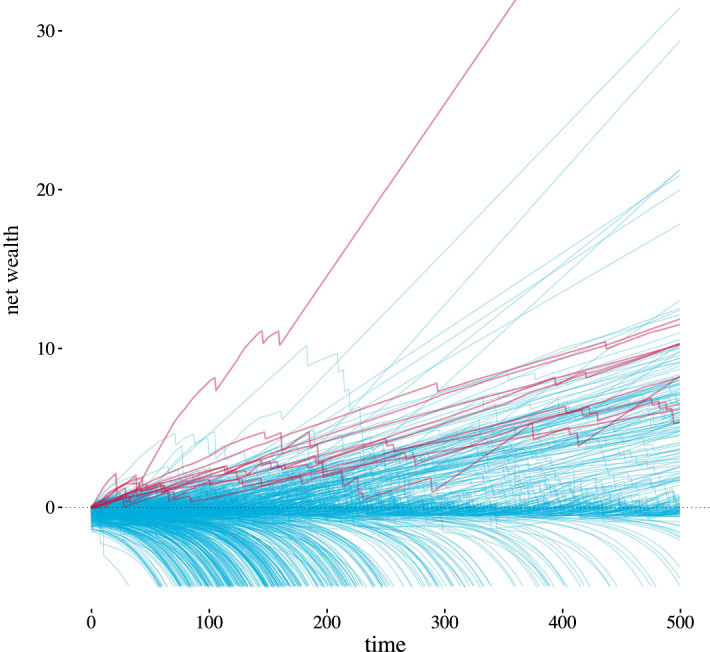
Each agent’s net wealth over time is shown here as a spaghetti plot for all 600 agents, with negative values truncated at ${\texttt {net worth}} = -\,5$. Trajectories indicated in red are those of agents who never borrowed to change employment

The aggregate loan amounts are so great they overwhelm the positive wealth values. We therefore consider a variant of the wealth metric, net wealth, which is the difference between the sum of all agent savings and all agent loans. Figure 5 demonstrates the net wealth values, truncated at ${\texttt {net worth}} = -5$, for all 600 agents in a single run. Agents who do not borrow at all over the 500 time steps are highlighted in red. Note that in this case the highest net wealth value belongs to an agent who did not borrow at any time, but this result varies with run, and it is common for the highest net wealth agent to have borrowed at one or more points.

The model restricts agents who can make loans to those who don’t currently have one, which is a simplification of lenders’ risk avoidance. We conducted a sensitivity analysis with a variant of the model whereby agents can take out loans up to ten times their current wage, which amounts to agents being able to make roughly ten job changes or being self-employed five times. The model behaviour is the same as the base model in that thwarts exceed changes, though this occurs later than in the base version, and the quantity of loans grows superlinearly. Once the interest rate for loans rises as well, the thwarts exceed changes in the same timeframe as the base model. (Results for these alternative loan schemes are provided in “Appendix A.2.”) A model version where an agent can make unlimited loans results in a scenario nearly identical to that of free movement, with the difference being gross quantities of debt.

### Loans

**Fig. 6:**
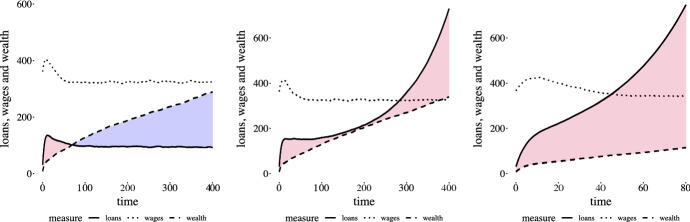
Loans, wages and savings over time for different savings rates. Plots of loans, wages and savings for values of lending rate 0%, 1% and 3%. Savings rates and cost multipliers are homogenous for all agents and types of moves, with values of 3% and 1. Net wealth, or savings minus loans, is indicated by the colored regions between the savings and loan lines. Blue indicates positive net wealth and red is negative net wealth

We noted in Sect. 3.4 that the total amount of loans in the simulated economies increases superlinearly for interest rates greater than 0. What is driving this superlinear behaviour? Figure 6 demonstrates model results over 30 individual runs for total loan amounts, wages and total savings for interest rates of 0% and 1% and 3%, with every agent having a savings rate of 3%. Cost multipliers for both moves and self-employment are homogenous with a value of 1. We see that with a lending rate of 0% there is no superlinearity in aggregate loan value and a positive net wealth.^13^ As seen in Fig. 4, the higher the lending rate the sooner the superlinearity appears. The colored regions indicate whether or not the difference between wealth and loans, or net wealth, is positive (blue) or negative (red). Superlinear behavior in loans in our costs with credit scenario results in total negative net wealth.

To explore the dynamics underlying this superlinearity in aggregate loan value, we consider $\lambda _1$ and $\lambda _2$ to be the quantity of loans at two consecutive time steps and if *l* is the lending rate, then $\lambda _2 = \lambda _1 + l\lambda _1 -$ loan payments $+$ new loans.

Loan payments are a function of the wages and savings rates of borrowers. If *s* is an agent’s savings rate and *w* his wage, and *b* the set of agents with outstanding loans, then loan payments are

3 $$\begin{aligned} \sum _bs_b\omega _b. \end{aligned}$$

where $s_b$ and $\omega _b$ are the savings rate and wage for borrower *b*. New loans are a function of the number of singleton loans, firm move loans, the costs for these two activities and the wages of the borrowers. If $c_s$ and $c_m$ are the wage multipliers to determine the costs for becoming self employed and changing firms respectively, and $\nu _s$ and $\nu _m$ the instances of new loans made to facilitate these activities, then the principle quantity of new loans are

4 $$\begin{aligned} \sum _{\nu _s}c_{s}\omega _{\nu _s} + \sum _{\nu _m}c_{m}\omega _{\nu _m}. \end{aligned}$$

Assuming mean wage $\omega$ represents any given borrower’s wage, mean savings rate *s* any given borrower’s rate, and mean costs multiplier *c* represents both singleton and move costs, and $\nu$ the total number of new loans, then Eq. 3 becomes $sb\omega$ and Eq. 4 becomes $c\nu \omega$, and the simplified total loan equation is

5 $$\begin{aligned} \lambda _2 = \lambda _1 + l\lambda _1 - sb\omega + c\nu \omega . \end{aligned}$$

The superlinear behavior is described by an increasing difference in consecutive $\lambda$ values. In the further simplified case of interest-free loans, $l = 0$ and if $\lambda _2 - \lambda _1 > 0$ then

6 $$\begin{aligned} c\nu \omega > sb\omega \end{aligned}$$

which reduces to

7 $$\begin{aligned} \nu c&> bs \nonumber \\ \frac{\nu }{b}&> \frac{s}{c}. \end{aligned}$$

Therefore aggregate loans will increase when the ratio of new loans to existing borrowers exceeds the ratio of savings to the wage multiplier for costs.

**Fig. 7:**
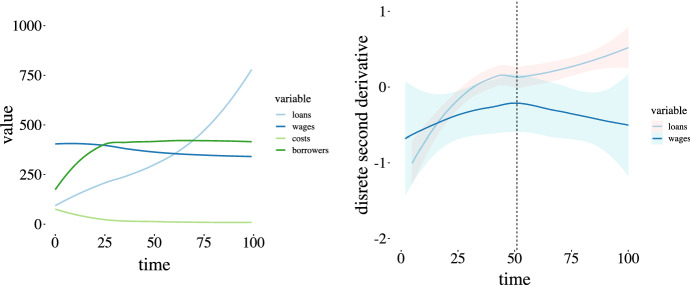
Loan parameters analysis. Simulation values averaged over 20 runs for the determinants of loan quantity with simulation parameters (left) and average discrete second derivatives of wages and loans (right). The cost multiplier for both startup and employer changes is 1, savings rate is homogenous at 3%. Note the correspondence of the inflection point

The plot in Fig. 7 on the left shows the simulation values for the elements in Eq. 7, costs, borrowers, wages and loans, with the lending rate 3% and homogenous savings and costs for both singletons and moves equal to 1.

The discrete second derivative of total loan and wage values for the two simulation are shown in the right hand plot in Fig. 7, and notice the matching inflection points in both the loan and wage curves around ${\texttt {t}} = 51$, which suggests a correlation between decreasing wages and increasing debt.

### Wealth Inequality

In the free movement and costs scenarios, all 600 agents have some amount of savings, while in the costs with credit scenario only 156 agents on average have savings greater than 0 at $t = 500$. The remaining 444 agents have debts, as illustrated in Fig. 5. Thus the model has produced a bimodal net wealth distribution roughly characterized by agents with debt and agents without debt, or agents with positive wealth and those with negative wealth.

**Fig. 8:**
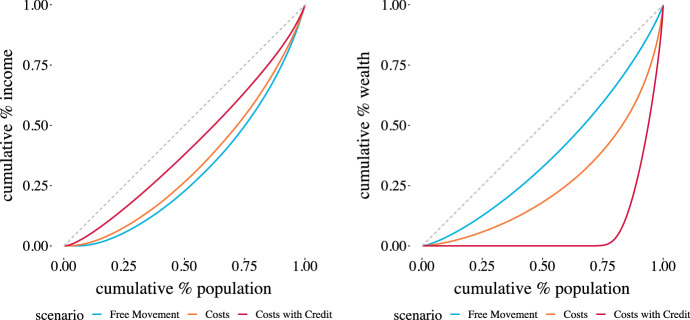
Income and wealth Lorentz curves for the three scenarios

Inequalities within a population are canonically represented by Lorentz curves, thus Fig. 8 demonstrates those curves for each of the scenarios for both wages (income) and total savings (wealth). It is interesting to note that while the three scenarios are not so different from the income perspective, they are hugely different from the wealth perspective, as demonstrated by the Gini Index values in Table 5. Note that the Gini values for income track with the wage variance for each of the scenarios.^14^

**Table 5:** Gini values for income and wealth distributions produced by model

Scenario	Income Gini	Wealth Gini
Free movement	39.4	24.7
Costs	33.8	48.8
Costs with credit	18.6	85.0

Gini value for income and wealth for each of the scenarios

## Discussion

The EF model demonstrates that adding cost constraints to free movement of workers, along with the ability to borrow to make a move, produces a negative feedback loop described by decreasing job mobility, savings and increasing debt, as hypothesized. As job mobility decreases in this costs with credit scenario, wages and savings fall and debt rises. As debt rises, job mobility and savings continues to fall and debt continues to rise, imitating the observed qualitative behaviours described in Sect. 1. The costs with credit model also produces levels of wealth inequality consistent with that observed in the US.

In the free movement scenario, agents are free to join firms until the optimal firm size is formed (Axtell 2018), at which point additional agents produce a free-rider effect that causes wages to fall and prompts agents to find better opportunities elsewhere. In this scenario both wage and size and wage and $\theta$ are most strongly correlated compared to other scenarios. In the costs scenario an agent must accrue enough savings to afford the costs of making a change, which adds a time delay in agent moves, roughly 30 time steps with a savings rate of 3%. By the time an agent can afford to move, their best utility options may have changed due to the movement of other agents and the constant reconfiguration of firms. Since thwarted agents include those who may want to leave a firm, firms in the costs scenario grow larger because agents are unable to leave. This impedance causes statistically significant increases in firm sizes, output and wages. Since agents aren’t free to move once a firm surpasses its optimal size and wages fall, the size-wage correlation decreases, but the size-$\beta$ correlation increases because mobile agents are attracted to highly productive firms with immobile high wage-preferenced workers.

Exploring the costs with credit scenario at the subpopulation level is highly informative. The debt-free subpopulation, the most mobile class, has the highest average firm $\beta$, but the lowest average $\theta$. Rather than high wage-preferenced agents grouping into highly productive firms, suggestive of the superstar firms discussed by Autor et al. (2020), agents move to more productive firms not because they produce more effort, but because they are mobile. This class also has the highest wage-$\theta$ correlation, indicative of the free-movement scenario. Conversely, the least mobile class, the subpopulation with increasing debt, has higher average $\theta$ values than the mobile class, but the lowest average firm $\beta$ value, suggesting that these agents are stuck in low-production free-riding firms. As in the costs scenario, this population also displays the strongest correlation between firm size and firm $\beta$.

In this model, as in the Axtell model, the firm production parameters *a*, *b* and $\beta$ are independent random variables and uncorrelated, and income preference is uncorrelated with an agent’s production parameters. This means that an above average $\beta$ value could be paired with a below average effort multiplier value, *a*, such that the two counteract each other in determining firm production. A future implementation could correlate these values for each agent, particularly the production parameters, to perhaps strengthen the productivity related correlations and further explore the emergence of agent homophily within firms under different scenarios.

Agent characteristics in this and the Axtell model are not dynamic, and network edges, effort preferences and productivity parameters are fixed. Currently, costs are modeled as a linear function of current wage, with the intent that an agent’s current situation is a representation of their network or capacities, but this means that lower wage agents incur lower mobility costs. Just as networks could evolve and become dynamic, the cost function could become nonlinear such that a low-wage agent could incur large mobility costs, representing an improving action such as upskilling, which would in turn update the agent’s productivity parameters.

In the costs with credit scenario a subset of agents are further impeded in making changes because they have outstanding loans they must pay off before they could either begin saving for a future move or borrow again. The superlinear growth in total loans exhibited in this scenario has multiple causes, all of which results in an agent not being able to save because they cannot pay off debt, and cannot move to an improved situation where they could earn higher wages in order to pay off debt. Savings rates are heterogenous in the EF model so there will be agents who make a loan and will not be able to repay that loan because their savings rate is lower than the lending rate. In another case, a perpetually indebted agent may have a saving rate equivalent to or higher than the lending rate, but may have chosen an opportunity that increased utility but decreased wage, again resulting in insufficient payments. Alternatively, an agent my have chosen a situation with a higher wage, but the decisions made by other agents eventually cause the firm’s productivity to decrease and the wage becomes insufficient to repay the loan. In each of these cases, the amount that borrowers owe will continue to grow over time. Unlike the costs scenario where there are two classes of agents, those with sufficient funds and those with temporarily insufficient funds, three classes of agents emerge in the costs with credit scenario: agents with sufficient savings who move at will, agents with loans who will pay off that loan and either borrow again to make a move or accrue savings before an opportunity arises, and agents who are hopelessly indebted and will never make a move. Changes are rare in the costs with credit scenario, and since fewer agents are able to place themselves in superior situations both wages and productivity are depressed.

The superlinear growth behavior in aggregate loan values is an intriguing result as the total amount of loans will exceed the total wealth in the model in the situations where a significant number of agents are unable to repay loans. The purpose of credit is to allow agents to complete contracts otherwise not obtainable, thus expanding markets, or in this case, permitting an employee to make a beneficial change and therefore increasing wages and productivity. Yet clearly too much available credit has significant negative consequences (Turner 2017). Russo developed a multi-agent model exploring the effects of household credit and found that using credit can smooth consumption over business cycles, but eventually the debt burden leads to inequality, thus there is a tradeoff (2016). Is it possible to say anything about viable credit regimes? Eq. 7 suggests that for cost multiplier of 1 and homogenous savings and lending rates, loans will increase only if the ratio of new loans to existing borrowers exceeds the saving rate. Exploring this double-edged effect of debt would be an intriguing avenue of study, and is relevant to current issues such as student loans.

While the above suggests the possibility of a sustainable credit regime, it is in no way prescriptive. The EF model is highly stylized, and not stock-flow consistent. Credit does not come from another agent nor are costs and interest paid to another agent, so the total wealth in the system is greatest for free movement, where it is equal to the cumulative output over the simulation run, and least for the costs scenario. This current formulation means any growth in output is the result of more efficient combinations of workers. If we were to apply an endogenous rather than exogenous lender then the debts held by agents would show up as credits held by others. In these cases of negative wealth the Gini index can be greater than 1, and inequality even more widely spread (Chen et al. 1982).

Despite its stylized nature, the model demonstrates that if pursuing the best productive opportunity incurs costs, then having financial capital matters. Without that capital, and especially in the presence of debt, agents are unable to participate in the economic activity of finding the most efficient uses of their labor. As the effective *N* decreases the options available to other agents become increasingly limited. Thus changes are fewer in the costs with credit scenario not only because perpetually indebted agents won’t make moves, but also because there are fewer opportunities for improvement for the mobile agents. Van Bavel describes this lack of access and diminished participation as a hallmark of the downward trend in historic cycles of the rise and fall of market economies (2019). Our current crisis of capitalism may actually be a lack of distributed financial capital. This the basis of the freedom argument in support of universal basic income (Widerquist et al. 2013). Studies of a project by GiveDirectly in Kenya that provides a monthly subsistence income over twelve years claim a significant portion of recipients use that money toward entrepreneurial ends (Lowrey 2017).^15^ The town of Aarhus, Denmark has implemented a program to give people seeking employment roughly $5000 to do whatever they needed to do to find or create a job, whether training, tattoo removal, new wardrobes, job hunting travel, or whatever their unique circumstances require (Urbact 2017).^16^ As mentioned in Sect. 1, other common mobility costs are relocation expenses or the need to cover gaps in health insurance or income. Costs may also arise in the form of externalities such as child or elderly care. These examples highlight that costs associated with job mobility are varied and individual, and are thus difficult to account for econometrically.

Hayek observed that when dealing with complex systems ‘the aspects of the events to be accounted for about which we can get quantitative data are necessarily limited and may not include the important ones’ (1975). Perhaps mobility costs aren’t visible as such at the macroeconomic level, but still play a decisive microeconomic role in determining an individual’s access to advantageous employment opportunities, which suggests a novel research direction to discover the microeconomic empirical evidence that drives the complex interaction between wages, savings and debt.

## Conclusion

We have developed a stylized model to explore the hypothesis that the observed decline in job mobility could be the consequence of a complex interaction between mobility, savings, wages and debt. We indeed found evidence of such a negative feedback loop, as well as evidence that this dynamic results in a level of wealth inequality on the same scale as we see today in the US. The EF model serves as a qualitative experiment that can generate testable hypothesis and justify efforts to generate datasets describing this dynamic which could be tested empirically. Before expanding the EF model in scale,^17^ we believe further modifications to the original Axtell model are required to better capture employment dynamics, namely reworking the utility function determining an agent’s work effort to take into account worker-discipline dynamics which would put a floor under possible effort values to accommodate subsistence or debt obligations (Bowles and Boyer 1988), allowing for dynamic modification of agent production values, as well as incorporating an evolving social network. We believe the resulting model could provide a sound basis for a future quantitative exploration of wages, savings, debt and firm size distributions.

## Figures and Tables

**Fig. 9:**
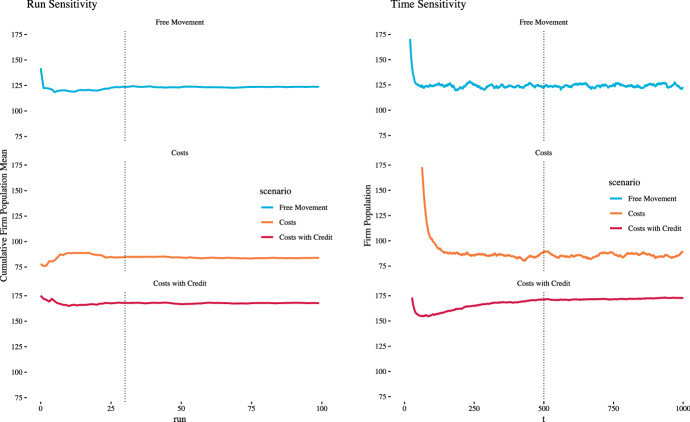
Run and time sensitivity

**Fig. 10:**
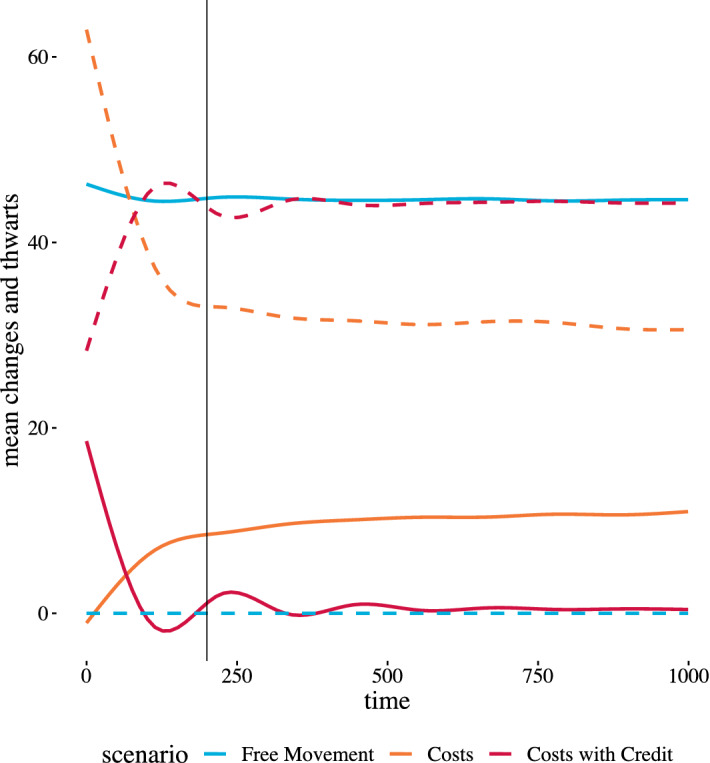
Change and thwart results for an alternative cost scheme where costs for changing jobs are based on expected wage rather than current wage

**Fig. 11:**
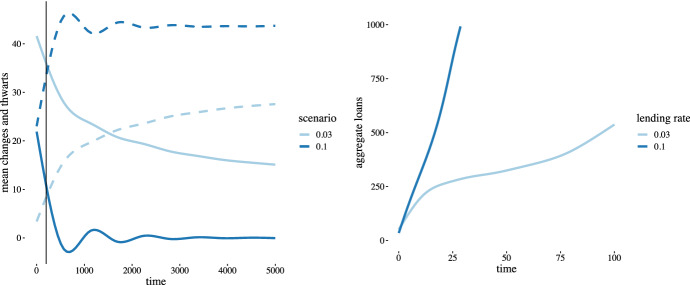
Aggregate loan amounts for two alternative loan schemes, the first with agents able to make loans up to ten times their wages with interest rate equal to savings rate, and the second where agents can take out loans up to ten times their wage, but with a lending rate of 10%

**Fig. 12:**
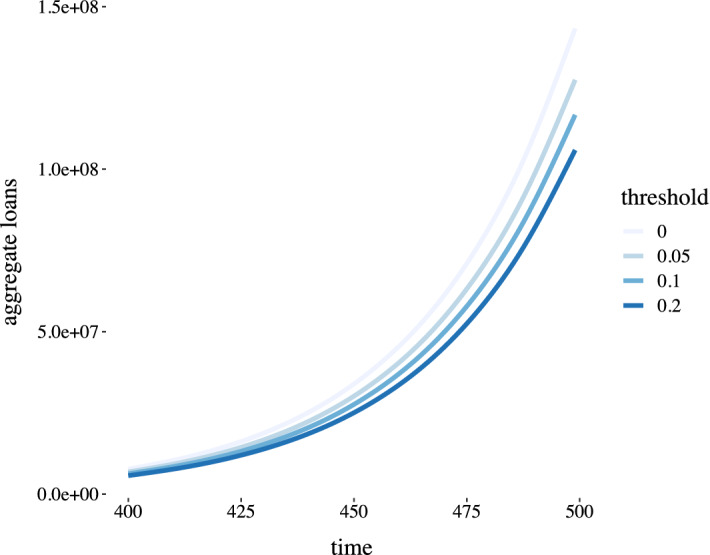
Aggregate loan amount persist in growing superlinearly even with minimum utility improvement thresholds

## A Sensitivity Analyses

### A.1 Run Parameter Selection

The EF model is a stylized small-scale model, and as such we minimized the runs, time steps and number of agents while still producing stable results in the free movement scenario. The plot on the left in Fig. 9 shows the cumulative number of firms over 100 runs, and our selected value of 30 runs is indicated by the dotted line. The values from ${\texttt {run}} = 30$ to ${\texttt {run}} = 100$ are statistically linear with a slope of 0. The plot on the right of Fig. 9 shows the number of firms over time out to 1000 time steps, and our selected values of 500 is likewise indicated.

The mean number and size of firms, the scaling factors for the distributions, and output and wages all scale linearly with N, thus we selected 600 agents as a computationally tractable number.

The model results are also independent of starting conditions, whether singleton firms, a single firm or starting anew with previous results obtained at ${\texttt {t}} = 100$. The results were very sensitive to the production parameters, with small variations in the range of values for the returns to scale exponent, $\beta$, producing changes in the mean firm size as well as the shape of the firm size distributions.^18^

### A.2 Cost and Loan Scheme Variations

In Sect. 3.1 we describe a sensitivity analysis where the cost of changing jobs depends on the expected wage instead of the current wage. Simulation results for for this analysis showing changes and thwarts for all scenarios is given in Fig. 10, which demonstrates that the pattern is the same as in the base model: fewer changes for the costs scenario compare to the free movement scenario, and even fewer changes for the costs with lending scenario.

As explained in Sect. 2, it is not our intention to model lending risk avoidance explicitly, so we used a simple lending scheme where interest rates matched mean savings rates, and borrowers couldn’t obtain new loans if they had one currently unpaid. In Sect. 3.4 we describe a set of sensitivity analyses exploring how alternate loan schemes affect the model outcomes. In the left-hand plot in Fig. 11 we show the results for the analyses where agents can borrow up to ten times their current wage with a lending rate of 3%, which is the same as the mean savings rate, and a lending rate of 10% (still significantly lower than modern consumer credit interest rates, which are upward of 20%). When an agent can borrow at will, the agent behaves as if it is in a free movement scenario, so the changes decrease and thwarts increase at a significantly slower rate than in the base model. However, when we raise the interest rate on this unsecured debt to 10%, the model behaves as the base model does with the number of changes quickly falling to low levels. In both cases, the aggregate amount of loans still increases superlinearly, as demonstrated in the right-hand plot in Fig. 11.

### A.3 Utility Improvement Thresholds

In the Axtell model, agents make changes to their employment whenever there is an opportunity to increase utility. If there are costs to making a change, it may be more reasonable to assume there is a minimum improvement in utility required before making a change. We ran the model for all scenarios with a series of increasing minimum improvement thresholds. For the free movement scenario, the number of changes decreases linearly with the threshold amount, such that the higher the threshold, the lower the changes, as expected. For the costs scenario, for a low threshold such as a 5% improvement, the number of changes actually increases slightly, before decreasing with increasing threshold values. This is because just a slight decrease in churn means more agents will have saved sufficiently before deciding to move, whereas this effect disappears for the larger reductions in churn caused by higher improvement thresholds. The costs with credit scenario produces linear decreases in both changes and thwarts. Figure 12 demonstrates that despite decreases in the amount of employment changes, aggregate loan amounts exhibit the same superlinear growth for the costs with credit scenario at least for threshold values up to 20%.

## Footnotes

1 Using a credit card qualifies as taking out a loan.

2 At the time of this writing, US economic activity has decreased and unemployment has risen dramatically due to novel coronovirus containment measures. These patterns held for pre-pandemic conditions.

3 Spatial mismatch theory suggests the ubiquitousness necessity of relocation in order to acquire ‘good ’ jobs (Andersson et al. 2018).

4 More broadly, Steindl explicitly modeled household saving and debt, and proposed that consumer credit could act as an economic stimulus (1990) whereas Dutt, on the other hand, finds evidence that consumer debt results in economic contraction (2006). If a decline in job mobility results in a decline in productivity, then we’d expect an economic contraction. This leads to the question of whether or not there exists a ‘right’ amount of consumer credit, which is intriguing but out of our current scope.

5 These economic patterns describe a pre-COVID-19 world.

6 Another way to express this idea is the *Matthew principle* whereby the rich get richer and the poor get poorer (Rigney 2010). This is related to the concept of preferential attachment (Barabási and Albert 1999) or preferential opportunity (Bottazzi and Secchi 2006; Arthur 1994).

7 Details of the Axtell model and the relationship between the Axtell and EF models can be found in Applegate (2018a).

8 Social networks could also be described by small world or preferential attachment structures (Watts and Strogatz 1998; Barabási and Albert 1999).

9 The Nash equilibria for this public goods game, with optimal firm sizes, is described by Axtell (2018).

10 The model code can be found in Applegate (2018b).

11 The emergence of this equilibrium band is a key characteristic of the Axtell model.

12 Classical productivity, total output divided by number of workers, is the same as mean wage in the model.

13 What happens in the cost with credit scenario when the interest rate is 0? Changes and thwarts mirror the costs scenario, with almost the same number of changes in the costs scenario as thwarts in the zero interest scenario, and the same number of changes in the zero interest scenario as thwarts in the cost scenario.

14 The World Economic Forum Inclusive Development Index for 2018 found the United States wealth Gini index to be 85.9. We find a value of 85.0 for the costs with credit scenario. We do not claim to be deriving the actual wealth Gini index, but the similarity demonstrates that the model generates levels of wealth inequality as extreme as we see empirically. In contrast, the income Gini for the United States according to the same source is 37.8. More sophisticated measures such as the Palma index reveal the structure of the inequality by explicitly comparing highest and lowest earners.

15 2020 Presidential candidate Andrew Yang’s *Freedom Dividend* is another such mechanism.

16 The Urbact case managers noted that an unexpected number of participants used the funds to become self-employed, and observed that there was no other mechanism available for people to obtain capital for such a purpose.

17 The Axtell model was run with 120 million agents to recreate 38 stylized facts about the US economy.
